# Intake of Table Sugar and Their Corresponding Food Sources in Adults from the 2017–2018 Brazilian National Dietary Survey

**DOI:** 10.3390/nu16071085

**Published:** 2024-04-07

**Authors:** Fábio da Veiga Ued, Paula Victória Félix, Carlos Alberto Nogueira-de-Almeida, Mauro Fisberg

**Affiliations:** 1 Department of Health Sciences, Ribeirão Preto Medical School, University of São Paulo, Ribeirão Preto 14049-900, Brazil; 2 Department of Nutrition, School of Public Health, University of São Paulo, São Paulo 01246-904, Brazil; paula.victoria.santos@usp.br; 3 Medical Department, Federal University of São Carlos, São Carlos 13565-905, Brazil; dr.nogueira@ufscar.br; 4 Instituto Pensi, Fundação José Luiz Egydio Setubal, Hospital Infantil Sabará, São Paulo 01227-200, Brazil; mauro.fisberg@pensi.org.br

**Keywords:** sugar, dietary sucrose, nutritional epidemiology, dietary survey, nutrient intake

## Abstract

Excessive intake of free sugars is associated with adverse health outcomes. Table sugar is one of the main dietary sources of free sugars; however, the amount added by Brazilian consumers in their culinary preparations is unknown. The aims were to estimate the daily intake of table sugar (g/day), its contribution to total energy intake (E%) and the main food groups that contribute to the intake of this sugar in a nationwide multi-ethnic sample of Brazilian adults (2017–2018 Brazilian National Dietary Survey). Based on two 24-h recalls adjusted for the within-person variation, the overall median table sugar intake was 14.3 g/day, corresponding to 3.2 E%. Males, individuals living in rural areas, with low income, low education and experiencing food insecurity had a higher intake of table sugar. The main food sources of table sugar were coffee (55.8%), juice (33.9%), milk-based preparations and smoothies (3.1%), powdered and processed juice (2.7%), whole milk (1.9%), and tea (1.6%). There are no recommendations regarding the limit of table sugar intake, but considering that the WHO limits the intake of free sugars to <10 E%, it is concluded that table sugar intake by Brazilians corresponds to about 30% of the upper recommended daily intake of free sugars.

## 1. Introduction

Systematic reviews show that excessive sugar intake is associated with adverse health outcomes, such as dental caries [1], weight gain [2], cardiovascular disease [3] and type II diabetes [4]. Chronic non-communicable diseases (NCDs) are responsible for around 74% of deaths worldwide [5] and represent a huge financial burden for health services and families in low- and middle-income countries [6], such as Brazil. Nutritional interventions for the treatment and prevention of these diseases are necessary [7], with reducing sugar intake being one of the proposed strategies [8].

Government and health authorities are responsible for establishing recommendations and guidelines regarding sugar intake. However, the term “sugar” is quite broad and needs to be well defined in order to establish public policies on intake guidelines. The definitions for total sugar, free sugar, added sugar and table sugar may vary according to different authors, which makes it difficult to monitor food intake and its effects on health [9,10]. For the purposes of standardizing the types of sugars mentioned in this paper, the following definitions will be adopted:

According to the Food and Drug Administration (FDA), total sugar includes sugars (mono- and disaccharides) naturally present in foods and beverages, such as sugar in milk and fruit, as well as any added sugars that may be present in the product [11]. To date, there are no daily intake recommendations for total sugars.

According to the World Health Organization (WHO), free sugar includes sugars (mono- and disaccharides) added to foods and beverages by the manufacturer, cook or consumer and sugars naturally present in honey, syrups, fruit juice and fruit juice concentrates [8]. This definition does not include sugar naturally present in milk, fruit or vegetables. The WHO recommends limiting free sugar intake to less than 10% (strong recommendation) or less than 5% (conditional recommendation) of total daily energy intake.

According to the European Food Safety Authority (EFSA), added sugar includes glucose, sucrose, fructose, starch hydrolysates (fructose syrup, glucose syrup) and other isolated sugar preparations that are added during manufacturing and food preparation [12]. The definition of added sugar does not include sugars naturally present in unsweetened fruit juice or honey [13]. The EFSA does not set a maximum intake limit for added sugar.

Table sugar is essentially pure sucrose [14] added to culinary preparations by the consumer. It is obtained by industrial processes, generally from sugar cane (*Saccharum officinarum* L.) or beet (*Beta vulgaris* L.) [10]. To date, there are no daily intake recommendations for table sugar.

The WHO has established recommendations on the intake of free sugars to reduce the risk of NCDs in adults, with a particular focus on the prevention and control of unhealthy weight gain and dental caries [8]. The recommended free sugar intake should be up to 50 g per day, based on a diet containing 2000 calories per day [8]. In 2021, the Brazilian Ministry of Health launched the 2021–2030 Strategic Action Plan for Tackling NCD in Brazil. In this document, one of the health promotion strategies aims to reduce sugar intake by encouraging changes in the nutritional composition of industrialized foods, promoting adequate labeling and communication campaigns [15] and emphasizing the need for free sugar intake below 10% of daily energy intake, as proposed by the WHO [8].

It is known that the high consumption of industrialized foods is responsible for the intake of free sugars above the WHO recommendation [16,17], but table sugar is also among the main dietary sources of free sugar [17]. However, the amount of table sugar added daily by Brazilian consumers in their culinary preparations is still unknown, which may contribute to the high intake of free sugars. Knowing this quantity and its associated factors can facilitate the adoption of public policies that contribute to the goal of reducing sugar intake by Brazilians by 2030.

This study aims to (i) describe the median daily intake (g/day) of table sugar, (ii) its contribution to total energy intake (E%), and (iii) the main food groups that contribute to the intake of this sugar by the Brazilian adult population.

## 2. Materials and Methods

### 2.1. Study Design and Population

Data from the Brazilian National Dietary Survey (BNDS) and the Household Budget Survey (HBS) were used [18,19]. These population surveys were conducted in 2017 and 2018 by the Brazilian Institute of Geography and Statistics (IBGE, Instituto Brasileiro de Geografia e Estatística). IBGE is the official Brazilian Population Statistics agency designed to collect data on the population’s living conditions, consumption expenditure and nutritional information in a representative sample of Brazilians.

**Figure 1:**
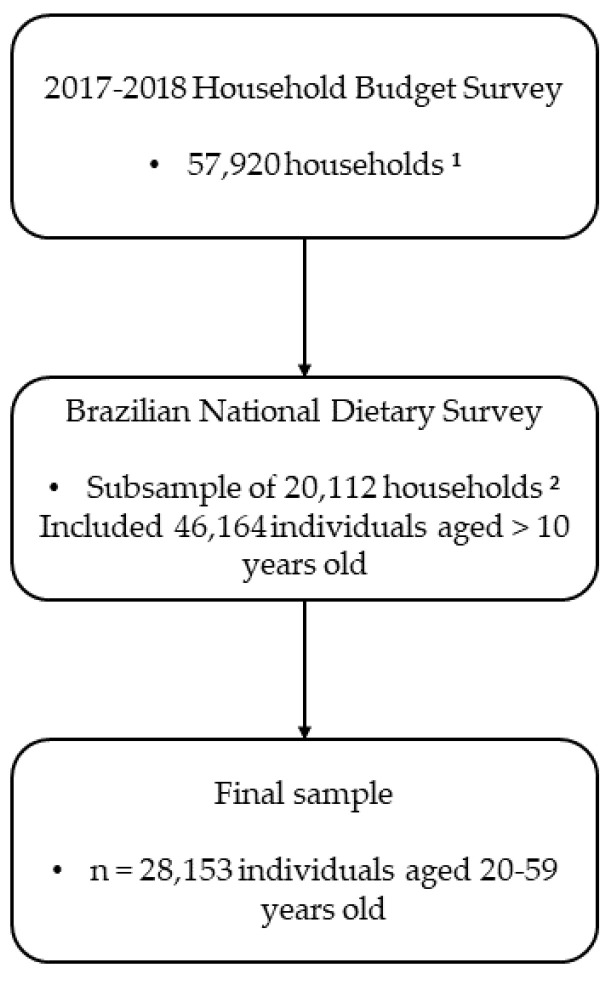
Sample flowchart in the 2017–2018 Household Budget Survey eligible for the present study. ^1^ Households selected at random from the pre-defined stratification system. An average loss of 15% was estimated due to possible refusals to answer the survey, and this same proportion was added to the final number of households to minimize possible losses. ^2^ Households selected at random from the pre-defined stratification system.

In HBS 2017–2018, participants were randomly selected through cluster sampling in two stages: census tracts (primary sampling unit) and households (secondary sampling unit). In the first stage, census tracts were stratified according to geographical (region, urban/rural areas, and administrative division) and household income [18]. A total of 5504 census tracts were selected, and sociodemographic, expenditure and life conditions information were collected from 57,920 households. In the second stage, a subsample of 20,112 households (~35% of the total sample) was randomly selected, including 46,164 participants aged >10 years, who completed at least one 24-h dietary recall (24HR) [19]. After removing adolescents, elderly people, pregnant women and breastfeeding women from the sample, the present study included a total of 28,153 adults (20–59 years, both sexes) with sociodemographic, life condition and dietary data collected (Figure 1). More details about the sampling plan can be obtained from IBGE documents [18,19].

The present study was conducted in accordance with the ethical principles of the Declaration of Helsinki. Furthermore, the guidelines of Brazilian Resolution Number 196/96, which discusses research involving humans, and Brazilian Law Number 5534 from 14 November 1968, which determines the confidentiality of information collected by all national censuses, were followed.

### 2.2. Sociodemographic and Anthropometric Information

The methodology for collecting sociodemographic and anthropometric information was previously published [20]. In general terms, the following information was collected by a structured questionnaire administered by trained interviewers at the household: sex, age, geographic region (North, Northeast, Midwest, Southeast, South), household area (urban/rural), self-reported ethnicity, self-reported weight and height, dietary habits, per capita family income, and education level (years of schooling). The body mass index (BMI) was calculated based on self-reported weight and height and classified according to WHO parameters [21]. Food security status was measured by the Brazilian Food Insecurity Scale (EBIA, Escala Brasileira de Insegurança Alimentar), an adapted scale from that proposed by the United States Department of Agriculture (USDA) and validated for the Brazilian population [22,23].

### 2.3. Dietary Data

Dietary data were collected using two non-consecutive 24HR, on all days of the week and seasons of the year. Both 24HR were collected by face-to-face interviews at participants’ homes following procedures described in the USDA Automated Multiple Pass Method [24]. Additionally, participants were asked whether they followed a specific diet for health purposes (e.g., for diabetes, hypercholesterolemia, obesity, hypertension or another health condition).

Individuals were advised by interviewers to inform them about all foods and beverages consumed in household measures and to report mealtimes, place of consumption (i.e., at home or away from home), cooking methods and seasonings added. The dietary data were entered into software developed by IBGE, which automatically converted home measurements into standard weight and volume measurements, such as grams and milliliters.

The energy and nutritional content of each food reported in 24HR was obtained from the Brazilian Food Composition Table (TBCA-USP), version 7.0, developed by the Food Research Center (FoRC) of the University of São Paulo (USP), available at http://www.fcf.usp.br/tbca (accessed on 15 July 2022), in accordance with standards and guidelines for the generation, compilation and use of food composition data from FAO/INFOODS (Food and Agriculture Organization/International Network of Food Data System).

Table sugar intake was adjusted for within-person variation using the web-based statistical modeling technique, multiple source method (MSM), version 1.0.1, updated in 2020. The MSM was developed within the framework of the European Food Validation and Consumption Project as a suitable technique for estimating the habitual intake of nutrients and foods (including those consumed sporadically) per individual based on two or more short-term dietary methods, such as 24HR [25,26]. The effects of the day-of-the-week (weekday vs. weekend) and atypical day of dietary intake (no vs. yes) were considered as adjustments in the models.

### 2.4. Food Grouping

The 1508 different foods reported in both 24HR were classified into 54 mutually exclusive food groups. The foods were combined based on frequency of consumption, similarity of nutritional profile, dietary habits and culinary use in the Brazilian population. Table sugar intake comprised sugar added by the consumer in culinary preparations.

### 2.5. Statistical Analyses

This report was prepared after strengthening the observational studies in nutritional epidemiology (STROBE-Nut) report specified for nutritional epidemiological studies [27]. Descriptive analyzes of median, percentage and 95% confidence intervals (95%CI) were performed using SPSS^®^ (version 22.0, 2013, IBM Corp., Armonk, NY, USA) and Stata^®^ (version 14.0, 2011, Stata Corp LP, College Station, TX, USA) software, considering a significance level of 5%. Descriptive statistics considered the complex sampling design of the National Dietary Survey 2017–2018 (svy family commands).

Differences in socioeconomic, demographic, anthropometric and lifestyle variables were tested by the Theil–Sen median test for complex sampling design. Dunn’s post hoc test was used to identify the significance between groups. The E% resulting from the daily intake of table sugar was estimated based on the average daily intake of 1795 kcal by the Brazilian adult population [19].

The organizational contribution of each food group to table sugar intake was determined using the method proposed by Block et al. [28]. This method estimates the corresponding percentage of foods or food groups consumed by the population out of total nutrient intake. Individuals who reported intake of a certain food group in at least one 24HR were classified as “consumers”, regardless of the amount reported. Consumer prevalence and table sugar content were calculated for food groups that contributed to >1% of total table sugar intake.

## 3. Results

### 3.1. Study Population Characteristics

A total of 28,153 Brazilian adults aged between 20 and 59 years participated in the study. The sample was predominantly composed of residents in urban areas (86.3%), in the Southeast region (42.8%), with a per capita family income > 1 minimum wage (40.4%) and with an education above high school (60.2%). Most of the population reported being black, mixed race or native (56.7%) and had excess body weight (55.6%). Regarding dietary characteristics, most participants reported not following a diet for weight loss or chronic disease treatment (86.9%) and having the habit of adding sugar to culinary dishes (79.7%). The EBIA classification indicated that a significant portion of the population experienced some degree of food insecurity (40.5%). Similar proportions were found for the variables of sex and age group. The sociodemographic, lifestyle and dietary characteristics of all included participants, the median intake (g/day) and E% resulting from daily table sugar intake are shown in Table 1.

### 3.2. Table Sugar Intake

The overall median table sugar intake of Brazilian adults was 14.3 g/day (95%CI: 3.7–31.8). As for the regions of Brazil, the lowest intake was found in the North region (8.6 g; 95% CI: 2.3–23.7) and the highest in the Northeast (18.9 g; 95% CI: 8.2–36.4). Individuals living in rural areas had a higher table sugar intake than those living in urban areas (16.5 g; 95% CI: 4.1–32.8 vs. 13.8 g; 95% CI: 3.3–31.7, respectively). In the analysis of age groups, there was a higher intake of table sugar among individuals aged 30 to 39 years (16.1 g; 95% CI: 4.0–32.9) and a lower intake among those aged 50 to 59 years (13.8 g; 95% CI: 2.9–31.5). The median intake of table sugar was higher in males (16.1 g; 95% CI: 3.9–32.9), in individuals who self-reported mixed race (15.7 g; 95% CI: 3.9–32.0), among those with a per capita family income below one minimum wage (15.9 g; 95% CI: 4.1–32.2), who had less than a primary education (15.9 g; 95% CI: 4.1–32.3), who had no excess body weight (15.4 g; 95% CI: 3.9–32.0), who reported not following a specific diet (16.1 g; 95% CI: 4.0–32.8), who declared the habit of adding table sugar to preparations of homemade cuisine (18.8 g; 95% CI: 8.2–34.2), and individuals classified as food insecurity status (15.4 g; 95% CI: 3.9–31.7).

### 3.3. Total Energy Intake (E%) Resulting from Daily Table Sugar Intake

Table sugar intake represented 3.2 E% in the diet of Brazilian adults. The highest percentage of energy resulting from the daily intake of table sugar was found in individuals who reported using sugar routinely and among residents of the Northeast region (4.2 E%), while the lowest percentage was observed among those who reported frequent use of sweetener (0.5 E%).

### 3.4. Main Food Sources of Table Sugar

The main food groups that contributed to table sugar intake are shown in Table 2. Coffee accounted for more than half of table sugar intake (55.8%), followed by juice (33.9%), milk-based preparations and smoothies (3.1%), powdered and processed juice (2.7%), whole milk (1.9%), and tea (1.6%), which together represented 99% of table sugar intake.

## 4. Discussion

This study revealed the amount of table sugar (sucrose) frequently used by the Brazilian population and the main food groups prepared at home that contributed to the intake of this sugar. It is not possible to say whether the median intake of table sugar by Brazilians (14.3 g/day) is high or low, as there are no national or international recommendations that establish an intake limit. In the adult populations of Portugal and England, table sugar intake represented approximately 6.8 and 30 g/day, respectively [14,17]. The use of 14.3 g of sugar to sweeten foods and drinks implies an increase of 57.2 kcal per day, resulting in 3.2 E%, considering the average energy intake of 1795 kcal/day by the Brazilian population.

Considering that the WHO recommendation limits the intake of free sugars < 10 E% [8], it is possible to verify that the intake of table sugar by Brazilians did not substantially contribute to reaching this limit, as it represented only 3.2 E%. On the other hand, if the WHO recommendation of <5 E% for free sugars is considered, the table sugar consumed by Brazilians exceeded 50% of this recommendation. There are authors who question recommendations that excessively limit the intake of free sugars, as they can reduce the intake of healthy foods rich in nutrients and from local culture [9]. In Brazil, the Food Guide for the Brazilian Population informs us that table sugar can be used in small quantities to create culinary preparations, contributing to diversifying the diet [29], despite not establishing a cut-off point for this consumption.

The prevalence of individuals consuming table sugar appears to be falling over the years in Brazil. Population-based research carried out in 2008 and 2009 [30] showed that the use of table sugar to sweeten food and drinks was reported by 90.8% of the Brazilian adult population, a proportion that decreased to 85.4% in 2017 and 2018 [19]. During this period, the preference to use neither sugar nor non-caloric sweeteners to sweeten foods and drinks increased from 1.5% to 6.9% of the adult population and the use of artificial sweeteners increased from 12.4% to 13.4% [19,30,31]. Despite this, it is not possible to conclude whether there was a reduction in the amount of table sugar ingested in g/day, as intake data were collected differently in the HBS editions. Other authors had already observed a trend of decreasing table sugar intake in Brazil in the first decade of the 21st century, but on the other hand, they observed an increase in the consumption of industrialized products containing added sugar [16].

Despite the reduction in the prevalence of table sugar use in Brazil, epidemiological studies have indicated an increase in the prevalence of diabetes in this population. According to the 2013 National Health Survey (PNS) [32], carried out in partnership with the Ministry of Health and IBGE, the prevalence of self-reported diabetes in Brazil was 6.2%; more recently, PNS 2019 recorded this proportion at 7.7% [33]. The PNS 2019 does not distinguish between types of diabetes, but it is known that 90 to 95% of diabetes cases are type 2 [34]. However, it should be noted that the increase in the prevalence of type 2 diabetes in Brazil is due to the higher prevalence of obesity [34]. This increase in obesity rates may (or may not) have as one of the causal components the excess intake of table sugar, which contributes to excessive calorie intake [35]. However, considering that the intake of this type of sugar has decreased in the Brazilian population, it cannot be said that table sugar is responsible for the increase in obesity rates and, consequently, diabetes. It should be noted that sucrose should not be prohibited in the diet of diabetics but restricted [34]. Despite this, public policies to raise awareness regarding the intake of free sugars must be permanent to avoid new health problems resulting from excessive sugar intake [36].

The use of sugar to sweeten foods and drinks is quite widespread in Brazil and it was observed that this habit was more practiced by men, individuals without excess body weight, residents of the Northeast region, residents of rural areas, citizens experiencing situations of food insecurity, and among individuals with low income and low education. The high intake of table sugar among men is consistent with the high proportion of unfavorable eating habits observed in this population group [37,38,39]. On the other hand, the lower intake of table sugar by individuals with excess body weight can be explained as an alternative to controlling weight gain and associated comorbidities. As for differences in the amount of table sugar consumed between Brazilian macro-regions, these may reflect the country’s known social and economic inequalities that are associated with the condition of food insecurity, as in the Northeast region [40]. In Brazil, food insecurity mainly affects individuals with low income and low education, living in rural areas, and is inversely associated with adherence to dietary patterns that involve the intake of fruit, vegetables and whole grains [41,42,43,44]. Among adults experiencing food insecurity, the prevailing eating pattern consists of white bread and toast, butter and margarine, table sugar, coffee and juice [41]. Food insecurity has a negative impact on the adoption of a dietary pattern rich in micronutrients and the absence of public policies contributes to a greater intake of foods and ingredients with lower nutritional value [41]. The higher intake of table sugar by the low-income population can also be explained by the presence of sugar in the Brazilian basic food basket (a set of foods provided by employers to meet the minimum needs of workers and their families). The average amount of sugar supplied in the Brazilian basic food basket is 3 kg per month [45]. This amount is equivalent to the intake of 100 g of sugar per day for a single person [45], but it should be noted that in many households, this product is shared among other family members, and, in some cases, the product may be discarded, which reduces per capita intake.

The dietary data detailed in the present study made it possible to identify the main dietary sources of table sugar consumed by Brazilian adults. The food groups that contributed most to total table sugar intake were coffee, juice, milk-based preparations and smoothies, powdered and processed juice, whole milk and tea. Coffee was the food most frequently mentioned in the HBS in 2017–2018 (78.1% of the population) and in 2008–2009 (79%), which refers to a very common habit of the Brazilian population, which is the consumption of coffee, white bread, butter/margarine and tea for breakfast [46]. These foods are typically consumed for breakfast by Brazilian adults, but if consumed exclusively, they may be indicative of a meal of low to moderate nutritional quality according to the Brazilian Breakfast Quality Index (BQI) [41,47]. The exclusive consumption of coffee without milk, white bread, butter and margarine were among the five most consumed foods by individuals with low to medium BQI indices [47]. Public policies aimed at minimizing food insecurity and encouraging healthy culinary practices for consumers should focus on this target audience.

Among the strengths of the present study, we can highlight: (I) the analysis of the most recent and available data on a representative sample of Brazilians, capable of providing updated evidence on table sugar intake and its dietary sources; (II) quality control in data collection; and (III) adjustment for intrapersonal variation in food intake data.

However, the present study has some limitations: (I) it was not possible to infer whether the amount of table sugar consumed is ideal, as there is no specific intake recommendation for this type of sugar; (II) it was not possible to assess the impact of table sugar intake on the consumption of total sugar, free sugar or added sugar as such information is not available so far in HBS 2017–2018; therefore, it was not possible to determine the prevalence of individuals who consume excess sugar and who are at risk of developing NCDs; (III) food intake was estimated through self-reported information, therefore, it is important to consider the possibility of measurement error, memory bias and incorrect reporting of energy and nutrient intake; (IV) weight and height were self-reported and probably underestimated the number of participants who did not have excessive body weight.

## 5. Conclusions and Final Recommendations

Table sugar intake by Brazilian adults corresponds to about 30% of the upper recommended daily intake of free sugars proposed by the WHO. The food groups that contribute to table sugar intake in this population are part of the country’s food culture. Public policies must continue to reinforce the need for moderation in the total intake of free sugars, ensuring permanent access to healthy and sustainable foods, expanding access to nutrition programs for vulnerable groups, reducing social inequalities and promoting education and food sovereignty actions to minimize food insecurity, promote health and prevent NCDs.

## Figures and Tables

**Table 1:** Median intake and total energy intake (E%) resulting from daily table sugar intake according to socioeconomic, demographic and anthropometric characteristics of the Brazilian adult population. Household Brazilian Budget Survey, 2017–2018.

	Total Population		Table Sugar Intake (g/Day)			
Characteristics	n	% (95% CI) ^1^	Median	IQR	*p*-Value ^2^	E% ^3^
Overall population	28,153	100	14.3	(3.7, 31.8)	-	3.2
Geographic region						
North	4132	8.2 (7.7, 8.7)	8.6 ^ab^	(2.3, 23.7)		1.9
Northeast	9717	26.4 (25.6, 27.3)	18.9 ^ab^	(8.2, 36.4)		4.2
Southeast	7029	42.8 (41.7, 44.0)	13.8 ^b^	(2.7, 13.8)		3.1
South	3699	14.6 (13.9, 15.4)	15.1 ^ab^	(3.9, 32.5)		3.4
Midwest	3576	7.9 (7.5, 8.4)	13.6 ^a^	(2.9, 28.4)	<0.001	3.0
Household area						
Urban	21,863	86.3 (85.7, 86.9)	13.8	(3.3, 31.7)		3.1
Rural	6290	13.7 (13.1, 14.3)	16.5	(4.1, 32.8)	<0.001	3.7
Age group, years						
20–29	6665	25.1 (24.3, 26.0)	14.2 ^c^	(3.4, 32.0)		3.2
30–39	7598	26.9 (26.0, 27.9)	16.1 ^abc^	(4.0, 32.9)		3.6
40–49	7274	25.1 (24.2, 26.0)	14.0 ^b^	(3.9, 31.5)		3.1
50–59	6616	22.8 (22.0, 23.7)	13.8 ^a^	(2.9, 31.5)	<0.001	3.1
Sex						
Male	13,338	49.8 (49.2, 50.4)	16.1	(3.9, 32.9)		3.6
Female	14,815	50.2 (49.6, 50.8)	13.8	(3.3, 30.6)	<0.001	3.1
Self-reported ethnicity ^4^						
Mixed-race	14,532	45.1 (44.0, 46.2)	15.7 ^ab^	(3.9, 32.0)		3.5
White	10,351	42.6 (41.4, 43.8)	13.8 ^a^	(3.3, 31.6)		3.1
Black	2963	11.2 (10.5, 11.9)	14.3	(3.6, 32.4)		3.2
Asian	145	0.6 (0.4, 0.9)	10.7 ^b^	(2.1, 23.7)		2.4
Native	141	0.4 (0.3, 0.1)	13.7	(2.9, 28.4)	0.001	3.1
Per capita family income ^5^						
≤1 minimum wage	13,218	40.4 (39.1, 41.7)	15.9	(4.1, 32.2)		3.5
>1 minimum wage	14,935	59.6 (58.3, 60.8)	13.8	(2.9, 31.6)	<0.001	3.1
Education level						
≤9 years of schooling (below elementary school)	12,702	39.8 (38.7, 40.9)	15.9	(4.1, 32.3)		3.5
>9 years of schooling (above high school)	15,451	60.2 (59.1, 61.3)	13.8	(2.9, 31.7)	<0.001	3.1
Body Mass Index						
Without excessive body weight	12,676	44.4 (43.4, 45.4)	15.4	(3.9, 32.0)		3.5
With excessive body weight	15,477	55.6 (54.6, 56.5)	13.9	(3.5, 31.7)	0.013	3.1
Followed a specific diet						
Yes	3778	13.1 (12.4, 13.8)	8.2	(1.8, 23.7)		1.8
No	24,375	86.9 (86.2, 87.6)	16.1	(4.0, 32.8)	<0.001	3.6
Frequent use of sugar/sweetener						
Sugar	23,257	79.7 (78.6, 80.7)	18.8 ^a^	(8.2, 34.2)		4.2
Sweetener	1899	7.8 (7.2, 8.5)	2.0 ^a^	(1.2, 3.9)		0.5
Sugar and sweetener	1362	5.6 (5.1, 6.2)	11.5 ^a^	(2.5, 27.3)		2.6
No	1635	6.9 (6.1, 7.8)	2.2 ^a^	(1.3, 5.0)	<0.001	0.5
Food security status						
Food security	15,878	59.5 (58.1, 60.9)	13.9	(3.3, 32.0)		3.1
Food insecurity	12,275	40.5 (38.5, 42.5)	15.4	(3.9, 31.7)	0.011	3.4

Abbreviations: CI: confidence interval; IQR: interquartile range; E%: total energy intake. ^1^ % considers the sample design of the study. ^2^ The median and IQR were described, and differences were assessed using the Theil–Sen test. Dunn’s post hoc test was applied to compare variables in three or more groups. ^3^ Total energy intake resulting from the consumption of table sugar, based on the average daily intake of 1795 kcal by the Brazilian adult population. ^4^ Twenty-one individuals did not declare their ethnicity. ^5^ One minimum wage was approximately USD 298 in 2018. Medians in the same variable with the same superscript letters (a–c) are significantly different (*p* < 0.01).

**Table 2:** Description of food groups that contributed to >1% of total table sugar intake, prevalence of consumers and food group table sugar density among Brazilian adults. Household Brazilian Budget Survey, 2017–2018.

Rank	Food Groups	% Total Table Sugar Intake	% of Consumers	Daily Food Group Intake (g/Day)	Food Group Table Sugar Density (g/100 g)
1	Coffee	55.8	87.9	215.3	14.1
2	Juice	33.9	43.7	383.4	20.8
3	Milk-based preparations and smoothies	3.1	6.2	306.1	13.2
4	Powdered and processed juice	2.7	6.4	367.3	12.8
5	Whole milk	1.9	7.5	241.4	6.2
6	Tea	1.6	9.5	822.5	4.4

## Data Availability

Data used in the present study are publicly available by the Brazilian Institute of Geography and Statistics, accessed on 25 January 2024 (https://www.ibge.gov.br/estatisticas/sociais/saude/24786-pesquisa-de-orcamentos-familiares-2.html?=&t=microdados). The code used in this study is available upon request.
